# A worldwide synthesis of handball research: regional identities, performance themes, and emerging scientific directions

**DOI:** 10.3389/fspor.2026.1923172

**Published:** 2026-09-03

**Authors:** Csilla Ildikó Filó

**Affiliations:** Department of Pedagogy and Psychology, Faculty of Cultural Studies, Teacher Training and Rural Development, Illyés Gyula Pedagogical Institute, University of Pecs, Pecs, Hungary

**Keywords:** athlete development, biomechanics, handball, injury prevention, machine learning, performance analysis

## Abstract

The relationship between regional scientific investment and international competitive achievement in handball has never been systematically examined. This scoping review addresses that gap by providing the first globally contextualised synthesis of contemporary handball research, drawing on 205 DOI-indexed, peer-reviewed, English-language studies published between 2020 and 2026 and identified through a PRISMA 2020-compliant search of four major databases (PubMed/MEDLINE, Scopus, Web of Science Core Collection, SPORTDiscus). Evidence was mapped across nine international research regions and six performance-science themes, and a novel Competitive Performance Index (CPI) was constructed from official IHF and EHF records to enable structured comparison between scientific output and senior men's competitive achievement across the same period. Regional analysis reveals four distinct science-performance relationship patterns. Scandinavia (CPI = 40; 16 studies) demonstrates convergence: sustained scientific leadership in injury epidemiology, neuromuscular risk profiling, and load monitoring co-evolves with four consecutive World Championship titles and Olympic gold. The Iberian Peninsula (CPI = 11; 39 studies, the highest volume of any region) and Central-Eastern Europe (CPI = 0; 30 studies) exhibit a leading-indicator pattern, in which scientific investment appears to precede competitive breakthrough by a temporal lag. The German-speaking region (CPI = 7; 28 studies) illustrates how frontier AI-driven performance analytics and youth championship success may function as competitive force multipliers. Western Europe (CPI = 15; 11 studies) demonstrates that strategically concentrated science sustains elite performance without high publication volume. Six thematic pillars structure the evidence base: biomechanics, injury risk and prevention, tactical and match performance, training physiology and conditioning, youth development and maturation, and technology-enhanced analytics. These domains are increasingly interdependent, reflecting a sport entering a phase of genuine scientific maturity. Critical structural gaps persist: approximately 74% of studies are male-only, North America and Oceania are almost entirely absent from the corpus, and the Technology-Enhanced Analytics category (5% of studies) substantially underestimates the true volume of technology-infused research due to the hierarchical classification rule applied. The CPI framework introduced here provides a replicable tool for examining science-performance alignment in handball and, with adaptation, in other team sports.

## 1 Introduction

Handball has evolved into one of the most physically, technically, and tactically demanding team sports worldwide, and its global competitive landscape has grown dramatically more complex over the past decade. The 2025 IHF Men's World Championship was won by Denmark for the fourth consecutive time, with Croatia, France, and Portugal completing the podium—a result that reflects not only athletic excellence but also the deep performance-science infrastructures that underpin modern elite handball (1). Across the six major senior men's competitions held between 2020 and 2026—the Tokyo and Paris Olympic Games, three IHF World Championships, and two EHF European Championships—a remarkably consistent pattern emerges: the nations that dominate the medal table are, with very few exceptions, the same nations that drive the global scientific literature on handball performance.

This convergence is not coincidental. High-performance sport at the national level increasingly depends on the systematic generation, translation, and application of scientific knowledge—from injury surveillance and biomechanical diagnostics to tactical modelling, load management, and youth development frameworks. Scandinavia leads both the podium (Competitive Performance Index, CPI = 40 across 2020–2026) and the scientific corpus in injury epidemiology, neuromuscular risk profiling, and load monitoring; the German-speaking region, despite modest historical World Championship results, has emerged as the global leader in AI-supported performance analytics and recently claimed Olympic silver (Paris 2024) and EHF EURO silver (2026); the Iberian Peninsula, which leads the scientific literature in volume (39 studies) and tactical research sophistication, has simultaneously seen Portugal reach its highest-ever World Championship placements. These patterns suggest that scientific investment and competitive achievement are associated in structured and regionally differentiated ways—raising a question that no previous review has addressed: does the global geography of handball science mirror, anticipate, or diverge from the geography of handball performance?

Despite the rapid growth of the evidence base, the knowledge base remains deeply fragmented. Most existing reviews focus on narrow topics—throwing biomechanics, ACL injury mechanisms, plyometric training adaptations, or match analysis—often within a single country or methodological tradition (2, 3). Regional research signatures have never been synthesised in a unified performance-science framework that also contextualises scientific output against competitive achievement. Scandinavian countries have produced large-scale epidemiological and neuromuscular studies; the Iberian Peninsula leads tactical, perceptual-cognitive, and game-analysis research; German-speaking countries have become hubs for sensor-based assessment and machine-learning applications; the Balkans maintain a strong tradition in strength, power, and explosive performance research; Central-Eastern Europe has developed comprehensive youth-development and anthropometric-profiling systems; Western Europe has generated influential work in performance modelling and coaching science; Arab-West Asia has demonstrated rapid growth in plyometric interventions and immunological responses; East Asia contributes physiological, epidemiological, and movement-specific adaptation studies; and South America has emerged as a centre for nonlinear sport pedagogy and youth development research.

The six-year window of 2020–2026 was selected deliberately. It captures the period following the 2019 IHF rule amendments that accelerated tactical and physical evolution, coincides with a documented surge in handball science publication output, and allows sufficient study accumulation in emerging regions for meaningful cross-regional comparison. The present scoping review is based on 205 peer-reviewed, DOI-indexed, English-language handball studies identified through a PRISMA 2020-compliant four-database search. Its specific aims are: (1) to identify major international research hubs and their characteristic scientific profiles; (2) to organise evidence into six core performance-science themes; (3) to introduce and apply a Competitive Performance Index (CPI) enabling structured comparison between scientific productivity and competitive achievement across regions; and (4) to propose directions for future interdisciplinary handball research.

## 2 Materials and methods

### 2.1 Study design

This work is a scoping review conducted in accordance with the PRISMA 2020 guidelines for scoping reviews (4). The protocol was not pre-registered. Scoping review methodology was chosen because the aim was to map the extent, nature, and geographic distribution of the available evidence across a broad, heterogeneous field rather than to synthesise quantitative effect estimates; accordingly, no meta-analysis or formal risk-of-bias assessment was performed.

### 2.2 Search strategy and information sources

A systematic search was performed across four electronic databases: PubMed/MEDLINE, Scopus, Web of Science Core Collection, and SPORTDiscus. A supplementary search was conducted in Google Scholar to capture recently indexed or grey-area publications, and reference lists of all included studies were manually screened. All searches covered the period 1 January 2020–31 January 2026. Three complementary search strings were applied across all databases:

1. “handball” AND (performance OR biomechanics OR physiology OR injury OR training OR maturation)
2. “team handball” AND (neuromuscular OR plyometric OR “tactical analysis” OR “load monitoring”)
3. “handball” AND (“machine learning” OR GPS OR IMU OR “computer vision”)

Boolean operators (AND, OR) were used throughout. No language, study design, or publication type filters were applied at the search stage; all filters were applied during subsequent screening. The complete search log is available from the corresponding author upon request.

### 2.3 Eligibility criteria

#### 2.3.1 Inclusion

Studies were eligible if they: (a) were published between 1 January 2020 and 31 January 2026; (b) were written in English; (c) contained a valid DOI; (d) included handball athletes ranging from youth to professional level; and (e) reported original, primary data on at least one of the following performance-science domains: biomechanics or movement analysis; injury epidemiology, risk factors, or prevention; match performance, tactics, or perceptual-cognitive processes; training physiology, strength/power development, or conditioning; anthropometry, biological maturation, or developmental pathways; or technology-assisted assessment (IMU, GPS/LPS, machine learning, computer vision).

#### 2.3.2 Exclusion

Studies were excluded if they: involved non-handball populations; lacked a valid DOI; were published in a language other than English; were themselves reviews, meta-analyses, or systematic reviews; were conference abstracts, theses, case reports, or non-peer-reviewed documents; reported purely clinical or surgical outcomes unrelated to sport performance; were exclusively sociological or historical without performance data; had insufficient methodological reporting to allow variable extraction; or were conducted under COVID-19 pandemic restrictions (primarily 2020–2021) where non-representative training conditions, cancelled competitions, or restricted facility access were documented or likely to have materially affected outcomes. This exclusion criterion was applied conservatively: only studies explicitly reporting COVID-19-related disruptions as a confounding factor, or studies conducted exclusively during lockdown periods (March 2020–June 2021), were excluded. Studies from 2020 to 2021 that reported unaffected training continuity were retained.

### 2.4 Study selection and screening

**Figure 1:**
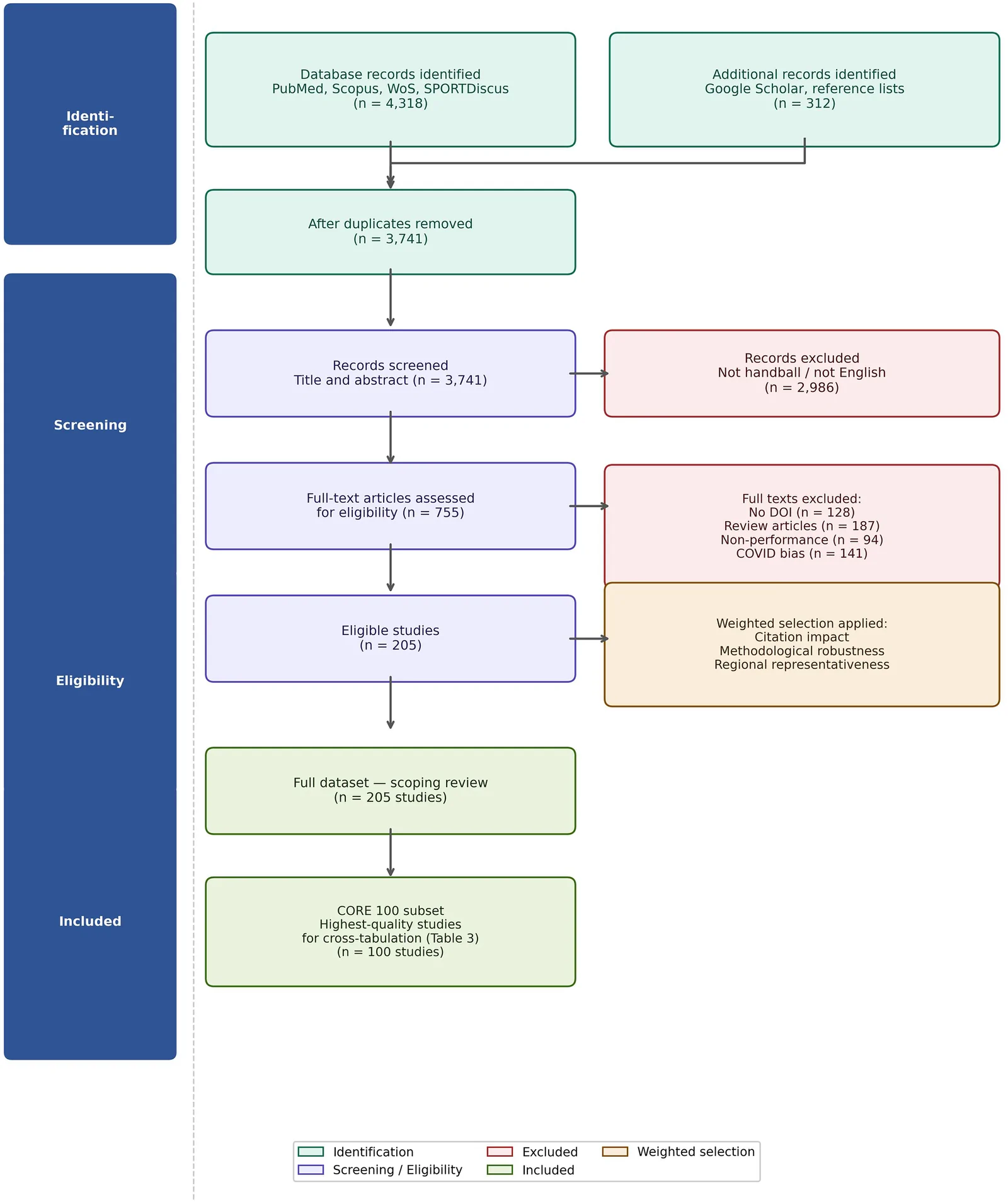
PRISMA 2020 flow diagram. Records identified: 4,630 | duplicates removed: 3,741 | screened: 3,741 | full texts assessed: 755 | excluded: 550 | included: 205 Figure 2. Regional scientific output vs. competitive achievement in handball (2020–2026). Bubble size proportional to CPI score. Dashed lines indicate corpus mean values. CPI = Competitive Performance Index (8 competitions, top-4 scoring). *N* = number of peer-reviewed studies in the corpus. Sources: IHF, EHF official records.

Study selection proceeded in three sequential stages, documented in the PRISMA 2020 flow diagram (Figure 1). In Stage 1, all retrieved records were screened by title and abstract for relevance to handball performance science. In Stage 2, full texts of potentially eligible articles were retrieved and assessed against the inclusion and exclusion criteria. In Stage 3, a weighted evidence selection model was applied to the 205 eligible studies to construct a representative rather than exhaustive synthesis, prioritising: (a) citation impact; (b) methodological robustness (larger samples, prospective or longitudinal designs, validated instruments, and more sophisticated analytic approaches); and (c) regional representativeness. Screening was performed by a single reviewer (C.F.); independent dual screening was not conducted, which constitutes a recognised limitation acknowledged in the Discussion.

### 2.5 Data extraction and regional classification

For each included study, the following variables were extracted: publication year; primary country of authorship and institutional affiliation; athlete population (sex, age group, competitive level); study design; sample size; performance domain(s); and primary outcomes. Each study was assigned to one of nine pre-defined global handball research regions: Scandinavia (Denmark, Norway, Sweden, Iceland); Iberian Peninsula (Spain, Portugal); German-speaking Region (Germany, Austria, Switzerland); Balkan Region (Serbia, Croatia, Slovenia, Bosnia-Herzegovina, Montenegro, North Macedonia); Central-Eastern Europe (Hungary, Poland, Romania, Czech Republic, Slovakia, Ukraine); Western Europe (France, Luxembourg, Belgium, Netherlands); East Asia (Japan, Korea, China); Arab-West Asia (Tunisia, Egypt, Qatar, Morocco, Jordan); and South America (Chile, Brazil, Argentina).

### 2.6 Thematic synthesis

Studies were assigned to one of six primary performance-science themes: Biomechanics and Motor Behaviour; Injury Risk and Prevention; Tactical and Match Performance; Training Physiology and Conditioning; Youth Development, Maturation, and Anthropometry; and Technology-Enhanced Analytics. When a study addressed multiple themes, a hierarchical decision rule was applied (biomechanics > injury > tactics > physiology > youth > technology). A weighted subset of 100 studies (CORE 100) was selected from the 205 eligible studies for use in the region-by-theme cross-tabulation (Table 3). The CORE 100 was constructed as follows: studies were first ranked within each of the nine regions by citation count (Google Scholar, accessed January 2026); ties were resolved by journal impact factor (JCR 2024); and within-region selection was capped proportionally [CORE N per region = round (region N/205 × 100)], ensuring that no region contributed more than its proportional share. The minimum allocation was one study per region. This procedure is reproducible from the study list available from the corresponding author. The implications of the hierarchical decision rule for thematic prevalence estimates are discussed in the Limitations section.

### 2.7 Competitive performance Index

To contextualise regional scientific output within the global landscape of handball competition, a Competitive Performance Index (CPI) was constructed for the study period (2020–2026). The CPI aggregates national team results across eight major senior men's international competitions held within this window: the Tokyo 2020 Olympic Games, the 2021 IHF World Championship, the 2022 EHF European Championship, the 2023 IHF World Championship, the 2024 EHF European Championship, the Paris 2024 Olympic Games, the 2025 IHF World Championship, and the 2026 EHF European Championship. Points were awarded using a standard descending podium system: 1st place = 4 points, 2nd place = 3 points, 3rd place = 2 points, 4th place = 1 point; no points were awarded for positions outside the top four. National scores were aggregated to the nine regional clusters defined in this review, with each nation's points counted independently.

Aggregated across all eight competitions, the resulting CPI scores were: Scandinavia (Denmark, Sweden, Iceland) = 40 points; Western Europe (France) = 15 points; Iberian Peninsula (Spain, Portugal) = 11 points; German-speaking Region (Germany) = 7 points; Balkan Region (Croatia, Slovenia) = 6 points; Arab-West Asia (Egypt) = 1 point; and Central-Eastern Europe, East Asia, and South America = 0 points each (no top-four finish within the study period). These scores are interpreted alongside each region's scientific output in the Results and Discussion.

Women's competitions were not included in the primary CPI for two reasons: first, the pronounced sex imbalance in the research corpus (∼74% male-only studies) renders cross-gender comparisons methodologically inconsistent; second, the women's competitive landscape exhibits a partially different regional structure (with Norway and France as dominant powers rather than Denmark). Women's competitive patterns are discussed qualitatively in the Discussion. The CPI is not a validated psychometric instrument but an exploratory descriptive tool designed to enable structured qualitative comparison between scientific productivity and competitive achievement across regions. All competition results were sourced from official IHF and EHF records; the full per-competition breakdown by nation is available from the corresponding author upon request. Scoring System Rationale and Sensitivity Analysis. The top-four threshold was selected because: (1) the top-four finish constitutes the universally recognised marker of elite competitive achievement in major handball tournaments, as it defines participation in the semi-final round and thus reflects sustained tournament performance; (2) it aligns with IHF and EHF official reporting conventions; and (3) extending beyond four placements would introduce substantial reliability concerns, as 5th–8th place differences are heavily influenced by bracket draw and qualification effects. The 4-3-2-1 linear scoring was preferred over exponential weighting to avoid artificially inflating single-competition winner dominance. All eight competitions were assigned equal weight; although the Olympic Games and IHF World Championships carry greater prestige, equal weighting was preferred to avoid arbitrary scaling choices, consistent with the exploratory nature of the index. To assess robustness, a sensitivity analysis was conducted using a prestige-weighted CPI (Olympics/WCH × 1.5 relative to EHF EURO). The weighted results were: Scandinavia = 57.5 pts, Western Europe = 21.0 pts, Iberia = 15.5 pts, German-speaking region = 10.5 pts, Balkans = 8.5 pts, Arab-West Asia = 1.5 pts, Central-Eastern Europe = East Asia = South America = 0. The regional ranking order is identical to the unweighted CPI, supporting robustness. Within-region asymmetry is acknowledged as a further limitation: within Scandinavia, Denmark accounts for approximately 33 of the 40 CPI points, reflecting four consecutive World Championship titles and Olympic gold; the Scandinavian CPI therefore primarily reflects Danish competitive dominance rather than uniform regional excellence. Similarly, France accounts for virtually all of Western Europe's CPI of 15 points. Readers are encouraged to interpret regional CPI scores with awareness of these underlying national distributions.

## 3 Results

### 3.1 Regional structure of global handball research (2020–2026)

The systematic search identified 4,630 records, of which 205 DOI-indexed, English-language, peer-reviewed handball studies met all eligibility criteria (Figure 1; Table 1). These studies originated from nine distinct regional hubs, each characterised by recognisable methodological signatures and thematic preferences. The CPI values presented below contextualise each region's scientific profile against its aggregate senior men's competitive achievement across the eight major international competitions held between 2020 and 2026 (see Methods).

**Table 1:** Regional distribution of included studies across the full dataset (*N* = 205) and the CORE methodologically robust subset (*N* = 100).

Region (Countries)	N Full Dataset (205)	N CORE Dataset (100)
1. Scandinavia (DEN, NOR, SWE, ISL)	16	8
2. Iberian Peninsula (ESP, POR)	39	22
3. German-speaking Region (GER, AUT, SUI)	28	10
4. Balkan Region (SRB, CRO, SLO, BIH, MNE, MKD)	24	12
5. Central-Eastern Europe (HUN, POL, ROM, CZE, SVK, UKR)	30	17
6. Western Europe (FRA, LUX, BEL, NED)	11	5
7. East Asia (JPN, KOR, CHN)	9	6
8. Arab-West Asia (TUN, EGY, QAT, MAR, JOR)	22	15
9. South America (CHL, BRA, ARG)	11	5

N, number of studies assigned to each region; CORE dataset, highest-quality, methodologically robust subset.

#### 3.1.1 Scandinavia (Denmark, Norway, Sweden, Iceland)—CPI: 40

Scandinavia leads both the competitive podium (CPI = 40, the highest of any region) and the global evidence base in injury prevention, longitudinal athlete monitoring, and neuromuscular risk profiling. Denmark's four consecutive IHF World Championship titles (2021, 2023, 2025) and Olympic gold medals are underpinned by one of the world's most developed performance-science ecosystems. Large-scale cohort investigations—most notably the HAPPY project involving approximately 945 youth players—provide fundamental insights into overuse injuries, growth-related vulnerabilities, and season-long health trajectories (5, 6). Icelandic studies examine training load interactions with overuse development during intensified preparation phases (7), while Scandinavian groups contribute ACL-related landing mechanics and neuromuscular risk profiling (8). GPS and LPS tracking support position-specific metabolic demand analysis, and goalkeeper anticipatory expertise is a recurrent thematic focus. The convergence of CPI dominance and scientific leadership is the most striking pattern in this review, suggesting a self-reinforcing cycle in which performance-science investment is associated with competitive success, and competitive ambition with further research investment.

#### 3.1.2 Iberian Peninsula (Spain, Portugal)—CPI: 11

The Iberian Peninsula presents the most scientifically productive region in the corpus (*n* = 39 studies) yet a CPI of only 11—a divergence that merits careful interpretation. Spain reached the top four in the Tokyo 2020 Olympics (bronze) and in the 2021 and 2023 World Championships (bronze), while Portugal achieved its best-ever World Championship result in 2025 (4th place). This trajectory suggests that Iberian science may operate as a leading indicator of competitive performance: the most extensive and sophisticated research output in tactical modelling, perceptual-cognitive decision-making, and throwing biomechanics (9–11) appears to be translating into improving competitive results with a temporal lag. The Iberian case thus offers a compelling illustration of science as a long-term investment in competitive infrastructure.

#### 3.1.3 German-Speaking region (Germany, Austria, Switzerland)—CPI: 7

The German-speaking region occupies a distinctive position: CPI = 7—reflecting Olympic silver (Paris 2024) and EHF EURO silver (2026)—combined with the third-highest scientific output (*n* = 28 studies) and the most technologically advanced research profile of any region. Machine learning, computer vision, and deep-neural-network approaches to tactical reconstruction define this region's scientific identity (12, 13), complemented by movement diagnostics evaluating deceleration mechanics, postural stability, and sensorimotor control. Notably, Germany's competitive resurgence—Olympic silver, EHF EURO silver, and the 2023 U21 World Championship title—coincides temporally with a sustained period of AI-driven performance analytics investment, consistent with the hypothesis that technological research investment may be associated with subsequent competitive advancement at senior level.

#### 3.1.4 Balkan region (Serbia, Croatia, Slovenia, bosnia-Herzegovina, Montenegro, north Macedonia)—CPI: 5

The Balkan region presents a growing competitive impact—Croatia's 2025 World Championship silver and EHF EURO bronze in 2026 represent the region's highest-ever collective senior podium contributions (CPI = 6)—not yet fully matched by publication volume (*n* = 24 studies). Croatian and Slovenian laboratories produce advanced analyses of jump-shot kinematics and 3D movement sequencing (14), while Serbian researchers have introduced novel conditioning paradigms including super-high-intensity continuous conditioning (15). The region emphasises strength, power, explosive movement, and throwing biomechanics—precisely the physical capacities that underpin elite performance.

#### 3.1.5 Central-eastern europe (Hungary, Poland, Romania, Czech republic, Slovakia, Ukraine)—CPI: 0

Central-Eastern Europe is the fastest-expanding scientific hub (*n* = 30 studies) yet holds a CPI of only 0, reflecting Hungary's 4th-place finish at the 2023 World Championship as the sole senior podium contribution. This extreme divergence is best understood through the level at which the science operates: the region's research focuses overwhelmingly on youth development, maturation, and anthropometric profiling (16, 17)—a long-cycle investment whose competitive returns appear most visibly at junior and U21 levels. Romanian and Ukrainian teams emphasise psychophysiological mechanisms including autonomic nervous system behaviour and stress reactivity (18). Hungary's 2023 U21 World Championship silver and the consistent presence of Central-Eastern European national academies in European youth competition suggest the CPI gap may close substantially over the next Olympic cycle.

#### 3.1.6 Western Europe (France, Luxembourg, Belgium, Netherlands)—CPI: 15

Western Europe achieves the second-highest CPI (15) despite the smallest scientific corpus among traditional European powers (*n* = 11 studies), reflecting France's sustained excellence—Olympic gold (Tokyo 2020), EHF EURO gold (2024), and World Championship silver and bronze. French research is characterised by big-data predictive modelling and coaching-psychology inquiry (19, 20) rather than high publication volume, suggesting a model in which concentrated, strategically targeted science supports elite performance without broad-base academic output.

#### 3.1.7 East Asia (Japan, Korea, China)—CPI: 0

East Asia has yet to reach the senior men's podium (CPI = 0) within the study period, yet its scientific activity is growing steadily (*n* = 9 studies). Japanese groups contribute insights into seasonal physiological variation and gut-microbiota dynamics (21); Korean scholarship provides prospective epidemiological investigations of elite players (22); and Chinese researchers produced the first handball-specific SSG meta-analysis (23). The zero CPI reflects a competitive gap relative to European powers rather than absence from global handball; the region participates regularly at World Championships and Olympiads. The scientific profile suggests a region investing in physiological and epidemiological infrastructure that may underpin future competitive development.

#### 3.1.8 Arab-West Asia (Tunisia, Egypt, Qatar, Morocco, Jordan)—CPI: 0

Arab-West Asia achieves CPI = 1—Egypt's 4th-place finish at Tokyo 2020—while producing 22 studies, the fourth-highest regional total. Applied intervention research—plyometric training, COD biomechanics, immunological monitoring, and nutritional periodisation—is advancing rapidly (24–26) without yet translating into top-four finishes. The 2025 U17 World Championship final (Germany vs. Egypt, with Morocco hosting) indicates genuine emerging-power status in younger age categories.

#### 3.1.9 South America (Chile, Brazil, Argentina)—CPI: 0

South America holds CPI = 0 and contributes 11 studies, yet its scientific identity is arguably the most distinctive of any region. Chilean, Brazilian, and Argentine research groups have pioneered nonlinear pedagogy, self-regulated training, and representative task design approaches now influencing handball coaching globally (27). The zero CPI reflects a structural competitive gap, yet the pedagogical innovations documented in the literature may represent exactly the kind of long-cycle developmental investment that produces competitive advancement over generational timescales.

### 3.2 Thematic structure of contemporary handball research

The 205 included studies were classified into six performance-science themes (Table 2). Biomechanics and Motor Behaviour was the most represented domain (*n* = 62, 30%), followed by Injury Risk and Prevention (*n* = 44, 21%), Training Physiology and Conditioning (*n* = 38, 19%), Tactical and Match Performance (*n* = 36, 18%), Youth Development and Maturation (*n* = 15, 7%), and Technology-Enhanced Analytics (*n* = 10, 5%). Tables 3, 4 cross-tabulate these themes by region.

**Table 2:** Thematic distribution of included studies across six performance-science domains for the full dataset and CORE subset.

Theme	N Full Dataset (205)	N CORE Dataset (100)
1. Biomechanics & Motor Behaviour	62	28
2. Injury Risk & Prevention	44	19
3. Tactical & Match Performance	36	14
4. Training Physiology & Conditioning	38	20
5. Youth Development & Maturation	15	12
6. Technology-Enhanced Analytics	10	7

Studies addressing multiple themes classified by hierarchical decision rule: biomechanics > injury > tactics > physiology > youth > technology.

**Table 3:** CORE 100 region-by-theme cross-tabulation showing study counts per cell.

Region	Biomech.	Injury	Tactical	**Physiol.**	**Youth**	Tech.	N
Scandinavia	2	4	1	0	1	0	**8**
Iberia	4	5	9	3	1	0	**22**
German-speaking	2	2	3	1	0	2	**10**
Balkans	7	1	1	1	2	0	**12**
C.-E. Europe	7	3	2	1	4	0	**17**
Western Europe	0	0	2	0	0	3	**5**
East Asia	1	1	1	2	0	1	**6**
Arab-W. Asia	3	3	0	8	1	0	**15**
South America	2	0	2	0	1	0	**5**

CORE 100, highest-quality subset; Biomech., Biomechanics; Physiol., Physiology; Tech., Technology.

The bold values in the N column represent the total number of studies in the CORE 100 dataset for each region, calculated as the sum of the six thematic categories in each row.

**Table 4:** Qualitative region-by-theme matrix: dominant thematic contributions of each research region (full 205-study corpus, 2020–2026).

Region	Biomechanics	Injury & Prevention	Tactical Performance	Training Physiology	Youth Development	Technology Analytics
Scandinavia (DEN-NOR-SWE-ISL)	Throwing mechanics, landing behaviour, neuromuscular control; eccentric force regulation	Global hub: epidemiology, overuse, ACL risk profiling; HAPPY cohort (n ∼945)	Goalkeeper anticipation; load-based perceptual analysis; position-specific metabolic demands	Extensive load, intensity and preseason readiness monitoring; GPS/LPS systems	Youth health monitoring models; integrated wellness and neuromuscular tracking	GPS/LPS used as measurement tool within biomechanical and injury studies; Tech = 0 in CORE 100a
Iberia (ESP-POR)	Upper-body kinematics; throwing determinants; anthropometric-kinetic chain interaction	Minor thematic presence; some overuse and ligament work	Most dominant region: offensive/defensive structures, decision-making, situational analysis; EHF and CL datasets	Internal load and recovery; caffeine and ergogenic aid research	Strong RAE and early specialisation evidence; talent identification critique	Advanced match and network analysis; situational modelling; AI-assisted tools
German-speaking Region (GER-AUT-SUI)	Deceleration mechanics; sensorimotor and postural control; movement diagnostics	Knee function, overuse patterns; cutting-technique risk analyses	Perceptual-motor and cognitive-decision studies; expert vs. amateur comparisons	Strength diagnostics; high-intensity conditioning	Coordination and perceptual learning; sensorimotor coupling in youth	World-leading: AI, deep learning, computer vision applied to tactical reconstruction
Balkan Region (SRB-CRO-SLO-BIH-MNE-MKD)	Elite throwing mechanics; CMJ profiling; COD mechanics; 2D/3D kinematic sequencing	ACL-linked biomechanical risk analyses; neuromuscular timing studies	Limited but emerging; tactical studies beginning to appear	Extremely strong: plyometrics, eccentric loading, super-high-intensity conditioning	Less represented; youth morphological profiling present	IMU-based kinematic measurement emerging
Central-Eastern Europe (HUN-POL-ROM-CZE-SVK-UKR)	COD-hip strength relationships; trunk mechanics; bilateral asymmetry assessment	Injury mechanisms in youth; overuse and growth-related vulnerability	Moderate presence; reactive agility and perceptual-motor coupling	Strength development and seasonal physiological adaptation; psychophysiological monitoring	Most dominant region: youth development, maturation profiling, anthropometry, RAE	Growing integration of sensorimotor and neuromuscular technology
Western Europe (FRA-LUX-BEL)	Limited emphasis	Limited emphasis	Coaching dynamics, tactical adaptation, big-data match modelling	Moderate; training load perspectives emerging	Minor; some RAE-adjacent developmental contributions	Strong: statistical modelling, predictive analytics, ML-based outcome prediction
East Asia (JPN-KOR-CHN)	Movement efficiency; sprint mechanics; meta-analysis on SSG training	Korea: robust prospective epidemiology of elite players	Limited tactical studies; growing analytical interest	Seasonal physiology, metabolic adaptation, gut microbiota dynamics	Emerging youth and developmental studies	GPS/sensor data integrated with physiological monitoring
Arab-West Asia (TUN-EGY-QAT-MAR-JOR)	High-quality COD, plyometric control, movement asymmetry	Strong immunological and inflammatory research (IL-6, TNF-alpha)	Growing interest in nonlinear pedagogy; contextual learning (MAR, JOR)	Strong intervention-based: plyometric, eccentric, sprint conditioning; Ramadan fasting	Increasing attention to youth female athletes; reference performance values	Early adoption of emerging analytic tools; Fitlight and reaction time systems
South America (CHL-BRA-ARG)	Limited emphasis	Limited emphasis	Distinctive: nonlinear defensive behaviour; contextual pedagogy; representative design	Self-regulated training interventions; ecological task constraints	Strongest region for pedagogical and youth skill-acquisition frameworks	Minimal technological applications; growing constraint manipulation interest

COD, change of direction; CMJ,countermovement jump; RAE, relative age effect; SSG, small-sided games; LPS, local positioning system; IMU, inertial measurement unit; AI, artificial intelligence; ML, machine learning. a GPS/LPS research is embedded within Biomechanics and Injury categories; Tech = 0 in CORE 100 subset (see Limitations).

#### 3.2.1 Biomechanics and motor behaviour

Hip-trunk rotational timing is consistently identified as a decisive determinant of ball velocity and shot accuracy, with elite athletes demonstrating more efficient kinetic-chain integration than sub-elite counterparts (14, 28). Advances in 3D motion capture and computer-vision applications have refined understanding of jump-shot mechanics and joint loading. Research on jump-landing mechanics and change-of-direction performance consistently identifies suboptimal landing strategies—excessive knee valgus, inadequate trunk stabilisation, and delayed neuromuscular responses—as associated with both decreased movement efficiency and elevated injury risk (8, 16).

#### 3.2.2 Injury risk and prevention

Epidemiological work from Scandinavia and East Asia documents overuse injuries, acute joint dysfunction, and season-long health problems across youth and elite populations (5, 6, 22). ACL injury remains the most extensively studied acute condition: athletes at higher risk exhibit pronounced knee valgus, decreased hip strength, insufficient trunk stabilisation, and delayed neuromuscular activation (8). Shoulder research identifies rotator-cuff imbalances and altered scapular mechanics as central contributors to overuse symptoms, with eccentric resistance training protocols appearing effective in restoring functional balance (29).

#### 3.2.3 Tactical and match performance analysis

Multi-season analyses reveal evolving offensive principles including increased positional interchangeability and emphasis on rapid transitions (9, 10). Machine-learning models predict match outcomes, classify tactical sequences, and estimate player contribution from tracking data (19). Defensive behaviour analysis highlights anticipatory expertise in goalkeepers and defenders, showing how experienced players use kinematic cues to predict and intercept actions (12, 13).

#### 3.2.4 Training physiology and conditioning

Research from the Balkans and Arab-West Asia provides substantial evidence for the effectiveness of plyometric, eccentric, and high-load resistance training in improving neuromuscular performance (15, 24, 25). Physiological studies document how metabolic strain, autonomic regulation, and inflammatory markers—including IL-6 and TNF-alpha—respond to training cycles and competitive loads (26). Ramadan fasting investigations and nutritional supplementation studies enrich the evidence base for culturally contextualised conditioning approaches.

#### 3.2.5 Youth development, maturation, and anthropometry

Research from Central-Eastern Europe demonstrates how biological maturation shapes strength, speed, throwing ability, and neuromuscular function, with growth-spurt periods associated with both performance gains and increased overuse vulnerability (16, 17). A pronounced Relative Age Effect in youth selection pathways is consistently identified, raising concerns about talent identification and long-term athlete development (30). Youth research increasingly integrates psychomotor and cognitive dimensions, aligning with nonlinear pedagogical approaches from South America (27).

#### 3.2.6 Technology-Enhanced performance analytics

Wearable IMU systems quantify movement intensity, asymmetry, and load in match-representative contexts (12). Computer-vision applications increasingly automate detection of throwing actions and tactical sequences, while AI-based models predict match outcomes and estimate player contribution with high accuracy (19). GPS/LPS tracking integrated with physiological monitoring—pioneered in Scandinavia and East Asia—provides comprehensive athlete readiness assessment, marking a shift toward data-rich, integrative performance analysis.

### 3.3 Region and theme matrix

**Figure 2:**
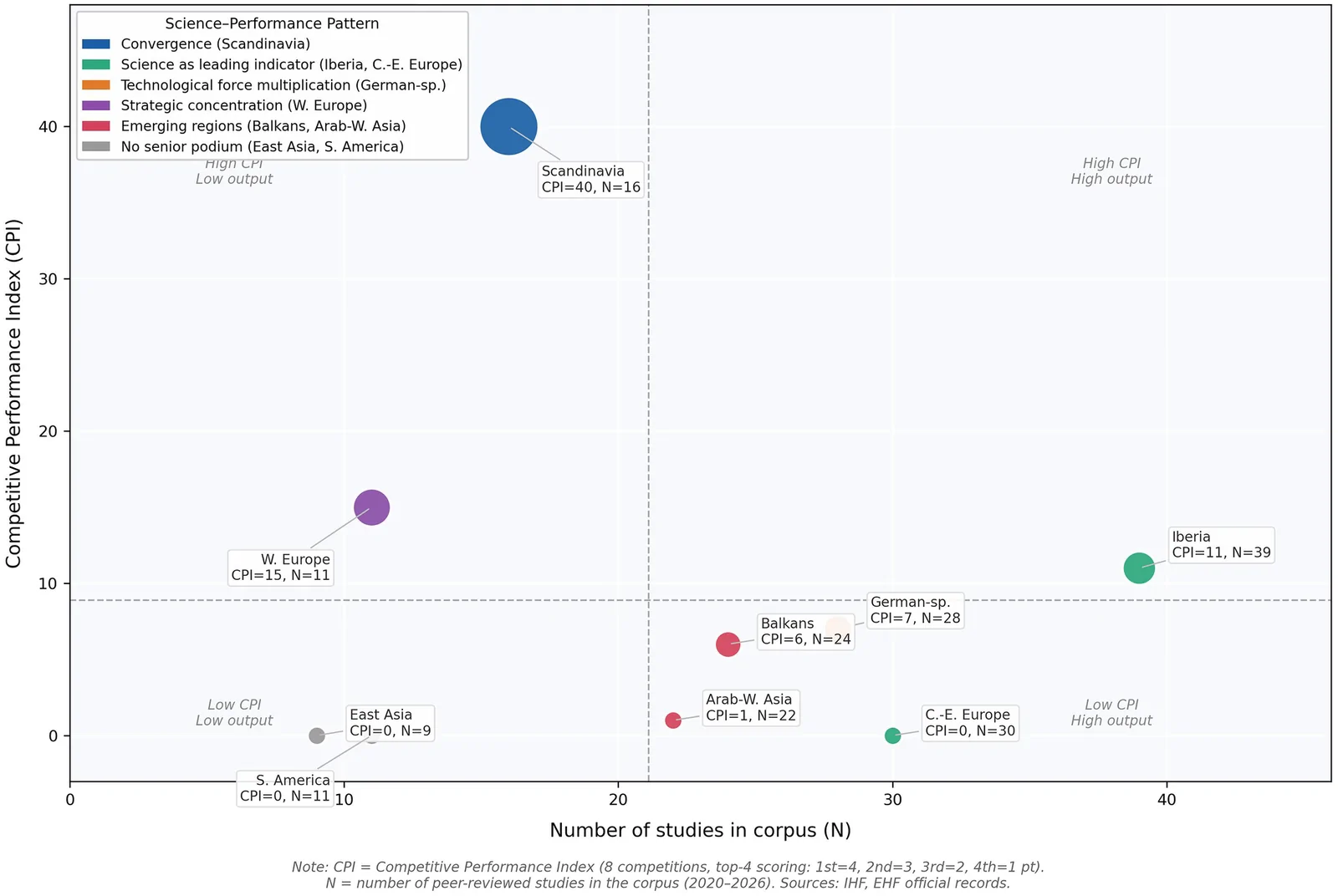
CPI scatter plot diagram.

Table 4 presents the qualitative distribution of thematic emphases across nine research regions. Scandinavia is the epicentre for epidemiology, neuromuscular biomechanics, and load-monitoring. Iberia dominates tactical and perceptual-cognitive research. The German-speaking region drives AI-supported performance analytics. Balkan research centres on strength, power, and throwing biomechanics. Central-Eastern Europe provides the most coherent body of evidence on youth development and maturation. Western Europe contributes predictive modelling and coaching science. East Asia is characterised by seasonal physiology and epidemiology. Arab-West Asia distinguishes itself through plyometric interventions and immunological responses. South America offers a distinctive pedagogical lens. Together, these nine regions constitute a dynamic, multipolar scientific ecosystem—and, as the CPI data reveal, one in which scientific investment and competitive achievement are associated in structured ways that no previous synthesis has examined. The relationship between scientific output (N studies) and competitive achievement (CPI) across all nine regions is visualised in Figure 2.

## 4 Discussion

### 4.1 Scientific output and competitive achievement: convergence, divergence, and the CPI framework

The most novel finding of this review is the structured relationship between regional scientific output and competitive performance as measured by the CPI. Across the nine regions examined, this relationship assumes four distinct forms that collectively challenge the implicit assumption that science and sport performance necessarily advance in lockstep.

#### 4.1.1 Convergence

Scandinavia (CPI = 40, 16 studies) demonstrates that sustained, systematically integrated performance science and podium dominance can co-evolve across decades. Denmark's four consecutive World Championship titles and Olympic gold are inseparable from the region's epidemiological surveillance infrastructure, neuromuscular diagnostics, and load-monitoring ecosystems (5, 6). Scandinavian national federation structures have formally institutionalised research translation, embedding sports scientists within national team environments in ways that directly inform selection, injury prevention, and periodisation decisions. Scandinavia thus represents the most well-documented example of science-performance co-occurrence in global handball.

#### 4.1.2 Scientific leadership as a leading indicator

Iberia (CPI = 11, 39 studies) and Central-Eastern Europe (CPI = 0, 30 studies) both produce substantially more science than their CPI scores might suggest is merited—but for different reasons. The Iberian case reflects tactical and cognitive research sophistication that appears to be translating into competitive results with a temporal lag: Spain's multiple top-four World Championship and Olympic finishes, combined with Portugal's breakthrough 4th-place World Championship finish in 2025, suggest that Iberian science may be functioning as competitive infrastructure in formation. Central-Eastern Europe presents an even more striking version: research overwhelmingly concentrated in youth development and maturation (16, 17) operates on a generational timescale, with Hungary's 2023 U21 World Championship silver signalling that the region's CPI may rise substantially in the next Olympic cycle.

#### 4.1.3 Technological breakthrough preceding competitive surge

The German-speaking region (CPI = 7, 28 studies) occupies perhaps the most intellectually interesting position. Germany's CPI—principally earned through Olympic silver and EHF EURO silver—does not reflect the region's position at the frontier of AI-driven performance analytics (12, 13). The German-speaking region produces the most technologically sophisticated research in the corpus, and Germany simultaneously claimed the 2023 U21 World Championship title. The temporal co-occurrence of technological research investment and competitive advancement at both junior and senior levels is consistent with the hypothesis that AI-driven performance intelligence systems may function as force multipliers in elite handball, enabling relatively rapid competitive advancement in systems that adopt them systematically.

#### 4.1.4 Competitive strength without proportional scientific investment

Western Europe (CPI = 15, 11 studies) achieves the second-highest CPI with the smallest scientific corpus among traditional European powers. France's extraordinary competitive record—Olympic gold, EHF EURO gold, World Championship silver and bronze—is sustained by a concentrated, strategically targeted scientific model rather than broad-base academic output (19, 20), suggesting that quality and strategic alignment of science may matter more than volume.

Together, these four patterns constitute a preliminary typology of science-performance relationships in handball that no previous review has articulated. The CPI framework, while exploratory and limited to senior men's competition, provides the first structured basis for cross-regional comparison of scientific and competitive development trajectories.

### 4.2 Thematic interdependencies and the architecture of modern handball science

Beyond regional patterns, the six identified themes form an integrated architecture rather than parallel silos. Biomechanics and injury risk form a unified mechanism: movement-pattern quality is central to both performance enhancement and vulnerability reduction (8, 14). Tactical decision-making is shaped by physiology and perception: fatigue accumulation and positional metabolic demands influence anticipation, cue recognition, and action selection under pressure (9, 12). Youth development acts as a gateway to all other themes: maturation status shapes neuromuscular capacity, injury susceptibility, and tactical understanding, while RAE studies indicate structural biases in early selection (16, 30). Technology acts as a cross-thematic catalyst: wearable sensors, computer vision, and machine learning create methodological bridges across biomechanics, physiology, tactics, and youth monitoring (12, 19, 23).

The most practically significant interdependency is between youth development science and long-term competitive achievement. The CPI analysis suggests that regions investing heavily in maturation profiling and youth-oriented conditioning research—Central-Eastern Europe most prominently—are building competitive infrastructure whose returns operate on a generational timescale. This represents a fundamentally different model of science-performance alignment from the acute load-monitoring and injury-prevention focus of Scandinavian research.

### 4.3 Gaps and structural asymmetries

Despite remarkable growth, several structural gaps persist. Geographically, Europe remains the overwhelming scientific stronghold: North America, Sub-Saharan Africa, and Oceania are almost entirely absent from the corpus, despite their growing presence in IHF competition calendars. The Arab-West Asian and East Asian regions, both CPI-limited but scientifically active, are best understood as emerging systems whose research investment precedes competitive breakthrough.

The sex imbalance is the most urgent structural gap. A quantitative analysis of the 205-study corpus reveals that approximately 74% of studies (*n* ≈ 152) were conducted exclusively with male athletes; studies focusing exclusively on female athletes account for approximately 23% of the corpus (*n* = 38), with a further ∼7% (*n* ≈ 15) employing mixed or comparative samples. This means that nearly three quarters of the current evidence base cannot be directly applied to female players without significant caution. Given documented sex differences in ACL injury mechanisms, throwing biomechanics, hormonal influences on training adaptation, and tactical role distribution, a sex-disaggregated CPI and parallel female corpus analysis represent the most important methodological extensions for future synthesis work.

Methodologically, the hierarchical decision rule (biomechanics > injury > tactics > physiology > youth > technology) systematically underestimates the true volume of technology-infused research. Sensor-based load monitoring, computer-vision analysis, and AI-supported motion capture appear throughout the Biomechanics, Injury, and Tactical categories without receiving Technology classifications—meaning the 5% Technology share in Table 2 is almost certainly a substantial undercount. Single-reviewer screening is a further limitation; the PRISMA flow diagram (Figure 1) makes the selection process transparent but does not eliminate the possibility of selection bias.

### 4.4 Practical implications for coaches, analysts, and federations

The CPI analysis yields practical implications extending beyond academic interest. Federations in regions with high scientific output but low CPI—Iberia, Central-Eastern Europe—should consider whether their research is being systematically translated into coaching practice, talent identification reform, and performance intelligence systems, or whether it remains primarily academic. The Scandinavian model—formal embedding of scientists within national team structures—provides the clearest template for research translation at elite level.

The German case suggests that AI-driven performance analytics and youth development science may represent disproportionately high-return investments for federations seeking competitive advancement. Germany's combination of U21 World Championship gold (2023), Olympic silver (2024), and EHF EURO silver (2026) emerged from a period of sustained technological research investment now visibly influencing senior team preparation. Load monitoring and athlete health surveillance remain foundational: Scandinavian epidemiological models demonstrate that systematic tracking of training load, overuse symptoms, and neuromuscular status is essential for sustaining competitive output across multi-year cycles. Finally, maturity-sensitive youth programming is not merely developmental best practice but a competitive strategy: Central-Eastern European research establishes that RAE correction in selection processes can expand the effective talent pool without reducing competitive standards (30).

### 4.5 Future research directions

Five priority directions emerge. First, longitudinal multi-centre youth studies across at least three regions would enable the first cross-cultural mapping of developmental archetypes, directly extending the CPI framework by tracking whether scientific investment in youth development translates into senior competitive advancement. Second, a sex-disaggregated parallel synthesis covering women's handball with its own CPI would address the most critical structural gap identified here. Third, technological standardisation enabling comparability between GPS/LPS and IMU systems is necessary before cross-regional load-monitoring benchmarks can be established. Fourth, integrative modelling studies linking biomechanics, physiology, cognition, and tactics within unified frameworks would advance understanding of performance as a systems-level phenomenon. Fifth, dedicated research programmes from currently underrepresented regions—North America, Sub-Saharan Africa, Oceania—are needed to test whether the science-performance relationships identified here generalise beyond the European-dominated landscape.

## 5 Conclusion

This scoping review provides the first globally contextualised, regionally differentiated, and thematically integrated synthesis of contemporary handball research, and the first to systematically examine the relationship between scientific output and competitive achievement across global research regions. Drawing on 205 DOI-indexed, peer-reviewed studies published between 2020 and 2026 and identified through a PRISMA 2020-compliant four-database search, the review maps handball performance science across nine international regions and six thematic domains—and introduces a Competitive Performance Index (CPI) that for the first time enables structured comparison between where science is produced and where medals are won.

The central finding is that the geography of handball science and the geography of handball performance are neither identical nor independent. Scandinavia's convergence of CPI dominance (40 points) and scientific leadership illustrates that sustained, institutionally embedded performance science and elite competitive success can mutually reinforce each other across decades. The Iberian Peninsula's position as the most scientifically productive region (39 studies) despite a moderate CPI (11) suggests that scientific investment operates as a leading indicator of competitive development. Germany's emergence as an Olympic and EHF EURO silver medallist concurrent with its frontier AI-driven analytics suggests that technological research investment may function as a competitive force multiplier. Central-Eastern Europe's extraordinary youth-science infrastructure against a near-zero senior CPI represents the longest-cycle form of science-performance alignment—one whose competitive dividends are most likely to materialise over the coming Olympic cycle.

Six thematic pillars structure the evidence base and increasingly intersect: biomechanics informs injury mechanisms; physiology shapes tactical execution; maturation determines neuromuscular capacity and injury vulnerability; technology bridges all domains as a cross-thematic catalyst. This architecture reflects a sport that has entered a phase of genuine scientific maturity—one in which knowledge is produced in integrated systems that mirror the multidimensional complexity of elite handball itself.

Critical gaps remain. The sex imbalance—approximately 74% male-only studies—means that nearly three quarters of the current evidence base cannot be applied to female players without significant caution, and the absence of a women's CPI analysis represents the most important limitation of this work. Geographic concentration in Europe leaves North America, Sub-Saharan Africa, and Oceania almost entirely absent. The broader implication of this review extends beyond handball: the CPI framework developed here offers a replicable, extensible tool for addressing the question of how—and how quickly—scientific investment translates into competitive return in handball and, with appropriate adaptation, in other team sports where regional research traditions and competitive landscapes can be similarly mapped.

## Data Availability

The full reference corpus (205 studies) is available upon reasonable request to the corresponding author.
